# A Neurophysiological and Neuropsychological Consideration of Mindful Movement: Clinical and Research Implications

**DOI:** 10.3389/fnhum.2015.00282

**Published:** 2015-05-26

**Authors:** Tamara Anne Russell, Silvia Maria Arcuri

**Affiliations:** ^1^Department of Psychosis Studies, Institute of Psychiatry, Psychology and Neurology, King’s College London, London, UK

**Keywords:** tai chi, mindful movement, default mode network, mindfulness, working memory, attention, psychosis, locus coeruleus/adrenaline

## Abstract

In this article, we present ideas related to three key aspects of mindfulness training: the regulation of attention via noradrenaline, the importance of working memory and its various components (particularly the central executive and episodic buffer), and the relationship of both of these to mind-wandering. These same aspects of mindfulness training are also involved in the preparation and execution of movement and implicated in the pathophysiology of psychosis. We argue that by moving in a mindful way, there may be an additive effect of training as the two elements of the practice (mindfulness and movement) independently, and perhaps synergistically, engage common underlying systems (the default mode network). We discuss how working with mindful movement may be one route to mindfulness training for individuals who would struggle to sit still to complete the more commonly taught mindfulness practices. Drawing on our clinical experience working with individuals with severe and enduring mental health conditions, we show the real world application of these ideas and how they can be used to help those who are suffering and for whom current treatments are still far from adequate.

## Introduction

Mindfulness in its modern secular manifestation has been defined as the “the awareness that emerges through paying attention on purpose, in the present moment, and non-judgmentally to the unfolding of experience” (Kabat-Zinn, 2003, p. 145). This training has its roots in centuries old traditions, yet is now delivered in standardized mindfulness training protocols (ST-Mindfulness)^1^ across a range of settings. This training in how to pay attention in a non-reactive, non-judgmental way is now well-established as beneficial for those suffering from chronic physical and mental health conditions (Fjorback et al., 2011; Williams and Kuyken, 2012; Khoury et al., 2013). However, one outstanding question relates to which specific components of this multifaceted intervention are contributing to the observed benefits (MacCoon et al., 2012; Williams et al., 2014).

A key training element is repeatedly attending to sensory information from the body. This is done through a variety of practices, which can be stationary (e.g., supine body scan) or moving (e.g., slow mindful walking, stretching, and yoga). The mindful movement practices may have a particular potency as evidenced by the finding that mindful yoga, although completed for the shortest amount of time relative to other practices, had the biggest impact on changes in mindfulness, well-being, and medical symptoms (Carmody and Baer, 2008). Mindful yoga practice time was also correlated with decreases in negative judgment of inner experience. Reduced judgment of inner experience contributes to changes in perceived stress in health workers (Shapiro et al., 2005) and reduced depressive symptoms in chronic depression (Kuyken et al., 2010). This indicates a strong rationale for mindful movement practices to be implemented more widely.

Most ST-Mindfulness practices require a certain degree of endurance in the ability to remain stationary for long periods, sometimes up to 45 min. This may be difficult for individuals who find it hard to remain seated and focused. Some of the groups who might therefore benefit from training based on mindful movement include: (i) those for whom the content and quality of mental experience is chaotic, disorganized, and distressing (e.g., visual or auditory hallucinations, paranoid delusions, racing thoughts and suicidal ideation, depersonalization); (ii) those whose illness may have compromised their attentional capabilities; (iii) those whose neurological or developmental disabilities make the metacognitive aspects of the training challenging; (iv) those for whom abnormalities (typically over-activity) in the motor system form part of the pathology of the disorder (for example ADHD, Tics, Tourette etc.); and (v) any others who find it hard to sit still for a relatively long period of time.

Mindful movement is a practice that may be of special relevance to those suffering from psychosis (and schizophrenia), an area of particular interest and specialization of the authors. High levels of stress are observed in these individuals. Not only do stressful life events predict relapse, there is also an effect of so-called daily hassles on relapse (Gispen-De Wied, 2000). One of the ways of coping with stress is to employ strategies. There is evidence that psychological interventions that deal with focusing attention and gaining control over mental experiences such as auditory hallucinations can reduce distress caused by symptoms (Shergill et al., 1998; Russell et al., 2008; Marsh et al., 2010). However, impaired cognitive performance may hamper this effort. There are a small number of studies using modified ST-Mindfulness protocols, and mindful movement with those experiencing psychosis (Russell, 2011; Chadwick, 2014), yet the wider delivery of mindfulness to these populations is hindered by beliefs that “mindfulness may be harmful for this client group” (Chadwick, 2014, p. 333).

In addition, the disorganized motor expression in this population has been neglected in current research. Often summarized and simplified under the term “agitation,” neuromotor signs are a key developmental feature of schizophrenia (Schiffman et al., 2004). Psychotic patients often present with severe imbalances in posture, co-ordination, and movement refinement before pharmacological treatment (Pappa and Dazzan, 2009), reflecting the disturbance of homeostasis in both body and mind. A more detailed examination of mindful movements for this population may thus help to advance the understanding of the pathophysiology of psychotic phenomena.

In this article, we present an argument for the utility of mindful *movement* as a mindfulness training methodology. We suggest some reasons why this training method may be particularly helpful for persons with *severe and enduring mental conditions* who struggle with ST-Mindfulness exercises. Our starting point was the clinical observation that mindful movements are reported by these patients as easier relative to static practices (Russell and Tatton-Ramos, 2014). Considering the intensity of their mind-wandering experience (Whitfield-Gabrieli and Ford, 2012), we have identified some mechanisms that intersect ST-Mindfulness training, movement practices, and the clinical picture of psychotic patients. These may share neural underpinnings, including those brain regions^2^ that are part of the default mode network (DMN), known to be disrupted in schizophrenia (Buckner et al., 2008; Hasenkamp et al., 2012; Whitfield-Gabrieli and Ford, 2012).

We are aware that we are proposing novel ways to look at phenomena observed both in mindfulness training and those suffering with psychotic symptoms. Our proposal offers some “pieces of a bigger puzzle,” with obvious gaps between some of the concepts discussed. It is beyond the scope of this article to propose a coherent and tested theory for the complex field of consciousness and its disturbances observed in psychosis. However, we believe mindful movement is a powerful intervention and it is our intention to open avenues for research and clinical work, and further elaborate the multiple routes to mindfulness training.

## Key Aspects of Mindfulness Training

A number of papers have proposed mechanisms underlying ST-Mindfulness and its effects [Shapiro et al., 2006; Teasdale and Chaskalson (Kulananda), 2011; Vago and Silbersweig, 2012], but scarce mention is made about these mechanisms with respect to the movement practices. While the *process* of mindfulness training is common to stationary and movement practices (paying attention, on purpose, moment-by-moment without judgment), the *object* (movement) has some distinct characteristics that may potentiate, as well as provide an alternative route to, mindfulness training.

Hasenkamp et al. (2012) defined four distinct phases to capture what occurs in the more basic type of meditation training (named focused attention): (i) mind-wandering; (ii) awareness of mind-wandering; (iii) an intentional shift of attention back to the object; and (iv) a sustained, focused attention on the object. They have suggested that mind-wandering engages the DMN, while the salience network (SN), and attentional networks/executive regions are engaged for the awareness of mind-wandering, the intentional shift of attention back to the object, and sustained focused attention (Hasenkamp et al., 2012). Mindfulness training results in a reduction of mind-wandering with a corresponding decrease in activation in the DMN (Brefczynski-Lewis et al., 2007; Pagnoni et al., 2008; Brewer et al., 2011).

Broadly speaking therefore, a key task in ST-Mindfulness is to monitor and regulate mind-wandering, by means of focusing attention on a chosen object. The basic features of this training are described in Section “Mind-Wandering.” This process requires regulation of the locus coeruleus^3^ (LC)/noradrenaline (NA)^4^ system (described in Section “Mind-Wandering and the Locus Coeruleus/Noradrenaline System”), and engagement of some of the components of the working memory system, specifically the central executive (CE) and episodic buffer (described in Section “Monitoring and Management of Mind-Wandering Requires Working Memory”). While these are only *some* of the components engaged in this complex mental training, they are particularly relevant here as they are also engaged in the generation and execution of movement (described in Section “Movements”). Thus, greater elaboration of these features may contribute to a further understanding of the mechanisms and effects of *mindful movement* practices. The commonalities between the neurophysiological and neuropsychological aspects of mindfulness training and movement, lead to a consideration of the additive effects of mindful movement (Section “Mindful Movements”).

### Mind-wandering

Mind-wandering and task focus are often treated as a dichotomy (Schad et al., 2012). When trying to attend to an object, there is more opportunity to notice mind-wandering. Although we are often largely unaware that we have mentally “wandered off,” one study indicates we are in this mode roughly 50% of the time (Killingsworth and Gilbert, 2010). The generic term “self-generated thoughts” has been proposed to capture this type of mental activity (Smallwood and Schooler, 2014). Mind-wandering can be intentional (e.g., planning, problem-solving, or reflecting), non-intentional (habitual), task-related or unrelated, and be of the “deep” (totally lost in thought) or “weak” (slight derailment) variety (Schad et al., 2012).

When attentional focus on the intended object wavers, there is an increased susceptibility to mind-wandering, and attention may be high-jacked by task-unrelated thoughts (TUTs). TUTs increase engagement with internal experience (including thoughts, images, memories) and reduce processing of external environmental events (Smallwood, 2013). The expression “lost in thought” describes the situation where we are no longer attentive to what is happening in the environment and are “up in our heads.” Being lost in thought could be a pleasant experience, for example, a daydream about a holiday. Alternatively, it may be unpleasant and distressing, for example, caught up in endless cycles of ruminative judging. A consequence of mindfulness training is an increased ability to recognize and “catch” mind-wandering and an improved ability to switch between states of mind-wandering and focused attention (Lutz et al., 2008).

### Mind-wandering and the locus coeruleus/noradrenaline system

Activity in the LC/NA system has been implicated in the occurrence of mind-wandering (as measured by TUTs, 27). The LC is a pontine structure, containing the largest number of noradrenergic neurons in the brain (Craven et al., 2005). It has extensive cortical projections, including to the pre-frontal cortex (Berridge and Waterhouse, 2003; Marien et al., 2004), an important feature which may underpin some of the cognitive effects of activity in this system (explained below). Employing two distinct modes of firing (phasic and tonic), the LC provides a range of arousal levels which optimize performance under different conditions (Smallwood et al., 2012). These patterns of firing modulate the system from states that are sleepy and dull to those of high arousal and excitability (see Table 1).

**Table 1:** **Locus coeruleus firing**.

	Sleep wake cycle mode				
	Awake			Asleep	
LC firing mode	Tonic		Phasic	Tonic	
Arousal state	Hypervigilant	Torpor	Variable (partly dependent on tonic)	Slow wave	REM
Firing pattern/NA level	High/increased	Low/decreased	Temporarily coupled to task-relevant processing	Low/decreased	Silent/decreased
Cognitive/mental state	Distractibility	Dream-like	Focused, selective attention, filtering out information that does not pertain to the task	?	Dreaming
Patients	High tonic at acute states (positive symptoms)	More prone to mind-wandering (negative symptoms)	Attention deficits, planning deficits, faulty filtering	Disturbed sleep	

During awake states, low (tonic) baseline firing levels are associated with drowsiness and torpor. High tonic baseline firing rates are associated with increased arousal and distractibility. Importantly, there is an interaction between those two modes (phasic and tonic) and the ability to focus attention (Berridge and Waterhouse, 2003). In a narrow range of tonic firing, it is possible for the phasic mode to occur. This phasic mode modulates the salience of information, which directly manipulates the attentional focus.

In the phasic mode, there are brief neuronal discharges within the LC. These discharges are coupled with the onset of the cognitive processing of task-relevant events, resulting in the amplification of the cortical representation of task-relevant information (Smallwood et al., 2012). Increased discharge of the LC, results in increased NA in the system. Thus, NA has been suggested as a chemical modulator of attention (Posner and Rothbart, 2007) mediating two functions: (i) the ability to select and attend to information that is relevant to the task and (ii) the ability to be alert to unexpected task-unrelated events (Craven et al., 2005). Such modulation serves an adaptive function and is necessary for survival. It facilitates the detection of sensory information relevant to a task, making salient (“popping out”) those stimuli that are aligned with the intention (see Box 1).

Box 1 **Searching for your keys without stress – importance of locus coeruleus/noradrenaline system.**

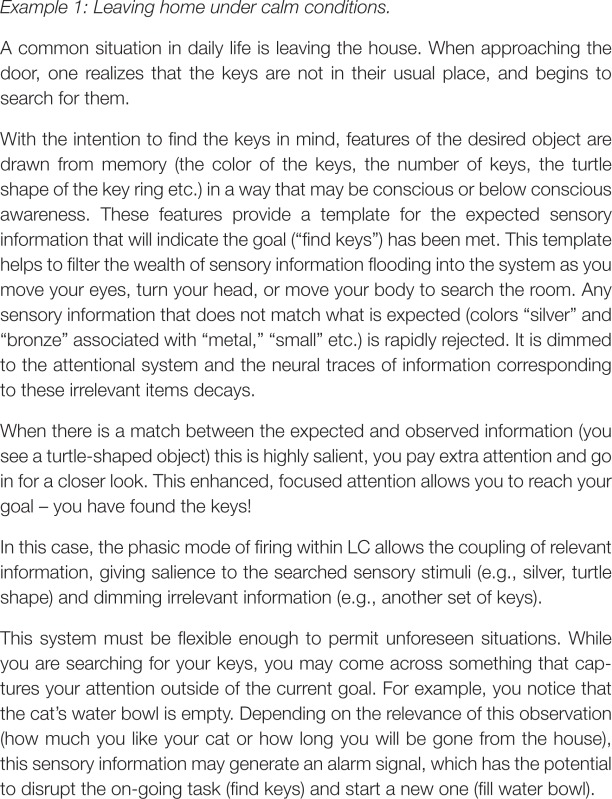

Increased tonic firing of the LC (with the resultant increase in NA in the system) disrupts this coupling process. Therefore, LC phasic responses become small or absent (Nieuwenhuis et al., 2005). This decoupling results in an “undifferentiated increase in cortical processing” (Smallwood et al., 2012, p. 2), meaning it is harder to distinguish between stimuli that are relevant and those that are not, nothing is “highlighted” or prioritized in terms of the attentional system (see Box 2). With increased NA activity, distractibility is thus magnified (Coull, 1994).

Box 2 **Searching for your keys with stress.**

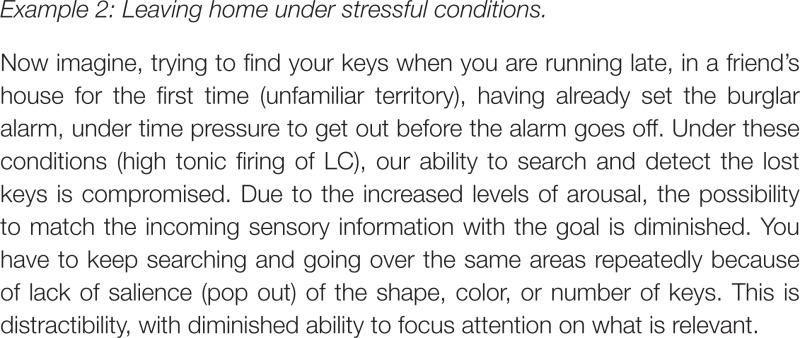

In mindfulness training, the task is to attend to the present moment (for example, attending to bodily sensations). The task requires an optimum state of “alert relaxation,” with the student monitoring the level of arousal. The student is also instructed to focus attention on specific information (e.g., sensations arising from the body). Mind-wandering occurs: (i) when distractibility is high and it is hard to keep the focus of attention and (ii) when in a more relaxed state, stimuli from the outside world become dimmed and the mind is carried away by thoughts, images, and dream-like states, leading to torpor.

In terms of the LC/NA system, these would respectively correspond to higher or lower tonic modes [outside the narrow range that allows the phasic bursts to occur (Berridge and Waterhouse, 2003)]. In both these situations, there would be a decoupling of the transient bursts, which are necessary to keep the body sensations salient. This means other task-unrelated information becomes equally salient. In mindfulness training, the detection of mind-wandering is a cue to renew the intention to attend to the object of choice.

As proposed by Hasenkamp et al. (2012), meditation training in focused attention requires the student to become aware of mind-wandering, to shift attention away from it, and to re-focus on the intended object. According to this description, selection of the incoming stimuli (body sensations as distinct from a range of other stimuli) should be temporally coupled with the intended goal (“attend to the body”). This would require modulation of the LC/NA system. One suggestion is that phasic activity would be coupled to the sensory information related to the task (e.g., sensations arising from the body). Coupled LC discharges (and corresponding NA bursts) with body sensations would then result in these becoming amplified, and all other sensations (e.g., the thought that captured attention and took us off task) dimmed.

The sections above have described the putative neurophysiological underpinnings important for mindfulness training, describing how the different modes of LC/NA firing relate to states of arousal, mind-wandering, and focused attention. From a cognitive point of view, in focused attention meditation practices three skills are required: (i) monitoring and vigilance to distractors whilst maintaining the focus of attention on an object; (ii) prompt disengagement or release from distractors; and (iii) deliberate re-focusing of attention back to the chosen object (Lutz et al., 2008). In the following sections, we describe the cognitive model which enables these distinct and complex functions required for ST-Mindfulness, via processes that have been ascribed to the CE and episodic buffer components of the working memory system (Baddeley, 1986).

### Monitoring and management of mind-wandering requires working memory

Working memory (and specifically its CE component) plays a key role in keeping clear priorities in the face of potential distractions (De Fockert, 2013). Therefore, during ST-Mindfulness, in order to keep the focus of attention whilst dealing with mind-wandering, such a system is very likely to be employed. A number of authors have implicated working memory in mindfulness training (Vago and Silbersweig, 2012) and its suggested therapeutic effects [Kerr et al., 2011; Teasdale and Chaskalson (Kulananda), 2011]. Experimental studies with both children (Schonert-Reichl et al., 2015) and adults (Jha et al., 2010; Mrazek et al., 2013) point to improvements in working memory capacity measured by neuropsychological tests following ST-Mindfulness. Under stressful conditions, short duration mindfulness training appears to reduce the deleterious effects of stress on working memory (Banks et al., 2015).

The term “working memory” falls within the broader construct of “executive functions,” defined by Luria [cited by Shallice (1982)] as a specialized system for the programing, regulation, and verification of activity involving the frontal lobes. More recently, the terms control, inhibition, and monitoring are used to describe these key executive functions. In mindfulness training, attention and working memory functions share common features (Buttle, 2011). There are also many overlaps between attentional and working memory systems in the brain (Nobre et al., 2004; Buttle, 2011; Gazzaley and Nobre, 2012). This can create confusion and a lack of specificity in the use of the term “working memory” (Vago and Silbersweig, 2012) may hinder research (Raz and Buhle, 2006).

The centrality of working memory in behavior (D’Esposito and Postle, 2015) and cognitive control (D’Esposito and Postle, 2015) has been demonstrated in research spanning over 50 years in the fields of experimental psychology and neuropsychology, as well as clinically. Although models of working memory continue to be refined and developed, we believe the specificity of the multicomponent model of working memory proposed by Baddeley and Hitch (Baddeley and Hitch, 1974; Baddeley, 1986) may provide a useful framework to help develop and refine cognitive models of mindfulness training [Shapiro et al., 2006; Teasdale and Chaskalson (Kulananda), 2011; Vago and Silbersweig, 2012]. This robust model has survived extensive experimental testing over many decades (Baddeley, 2002, 2012).

Within Baddeley’s framework, working memory comprises a CE with three auxiliary (slave) systems including the phonological loop, the visuo-spatial scratchpad (VSSP)^5^, and the episodic buffer. Baddeley has stated that “*the episodic buffer can be accessed by the CE via the medium of conscious awareness*” (Baddeley, 2000,p. 421), making these two parts of this model particularly relevant to mindfulness training.

The CE co-ordinates information processing with the help of these auxiliary systems. Baddeley described CE as “*an attentional control system with no intrinsic storage capacity”* (Baddeley, 2000, p. 420). It maintains intention (goals), monitors conflict, has a number of attentional roles (switching between tasks, focused attention, and divided attention) and interacts (via the auxiliary systems) with long-term memory. All these activities are coordinated to keep us “on task.” In ST-Mindfulness training, the CE is proposed to play a role in maintaining the overarching intention (e.g., “present moment awareness”) and to guide the attentional system (e.g., “attend to breath”).

The episodic buffer is described as a limited capacity, temporary, multimodal, storage system, which is at the interface between long-term memory and both the VSSP and phonological loop slave systems (Baddeley, 2000). The term “episodic” refers to a mode of operating, in which information is gathered together to form “chunks” or “episodes.” The term “buffer” refers to the characteristics of this system to maintain information temporarily on-line in order to manipulate it, transforming different types of information from a variety of systems into a common multidimensional code.

Thus, when performing mindfulness training, we propose it is the CE which allows us to become “conscious aware” (as Baddeley describes it) of the contents stored in the episodic buffer (breath or not breath, the latter being mind-wandering) and to reallocate attentional resources back to the intended goal (breath).

To summarize, mindfulness training develops the ability to modulate between states of alertness and sleepiness, and the capacity to monitor mind-wandering and focus attention. It develops cognitive “top-down” control over mind-wandering and focused attention via the CE of the working memory system. It also engages the episodic buffering function to hold on-line multimodal information and allow it to be manipulated in the service of higher order intentions (and behavioral control).

In addition to the role of working memory, in Section “Key Aspects of Mindfulness Training,” we have highlighted how the regulation of the LC/NA system is associated with mind-wandering which can interfere with the manipulation of attentional focus. We thus suggest that these two aspects are crucial to a better understanding of the mechanisms underlying the training of the focus of attention required in mindfulness meditation. In the following sections, we review evidence that moving the body engages these same systems (LC/NA and WM). What follows is not an exhaustive review of the motor sciences literature, rather we point to areas of overlap between motor movements and mindfulness training.

## Movements

### General cognitive aspects of movement

A topic of long-standing interest to researchers studying both attention and movement is the variable ways in which attention can be brought to action, and the discovery that it is possible to attend to movement in a variety of ways and at different levels of conscious awareness (Norman and Shallice, 1986; Frith, 2002). Norman and Shallice (1986) model of the supervisory attentional system (SAS) aimed “to produce an explanation for the different types of experience one can have of an action” (p. 14). They described five instances where it might be necessary to bring attention to a motor task (see Box 3). In such situations, the SAS, interacting with attention, selects and co-ordinates the desired response motor sequence (Badgaiyan, 2000). Baddeley integrated the SAS into his working memory model, naming it the CE (Baddeley, 2012). It is the same mechanism, with two different names. It is a control system that can be used to manipulate the contents of working memory storage in order to guide behavior in an effective way and particularly so when flexible responding is required (D’Esposito and Postle, 2015).

Box 3 **Five instances when it might be necessary to bring conscious attention to a motor task.**

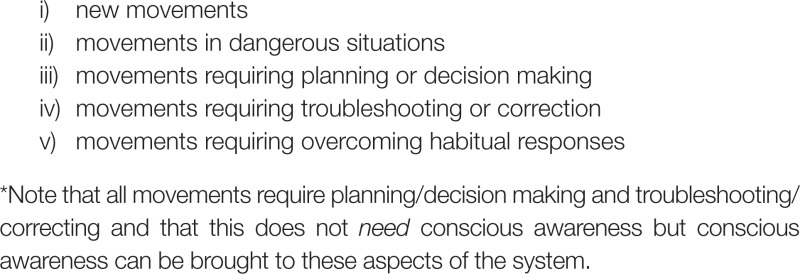

#### Automatic and Controlled Movement

The majority of movements made are highly automated and do not require any attention at all. Even a complex movement, like driving, can be done with little overt attention to the task (i.e., automatically). In this example, the mind is free to wander. However, it is possible to shift between automatic and controlled modes (in both directions). We argue that this process of shifting is inherent within the motor system, and in ST-Mindfulness occurs within an abstract mental realm. In ST-Mindfulness training, there is an intention to observe and undo habitual (automatic) mental reactivity in order to create new, healthier habits of responding (Kang et al., 2012). Thus, being able to shift between the automatic and controlled modes, via engagement of attention is important when training mindfulness. Moreover, this is also an intrinsic element of motor behavior, particularly when learning a new motor skill. The three-stage theory of motor learning proposes that after passing through the “cognitive” and “association” stages, we reach a third “automatic” stage (Fitts and Posner, 1967). In this automatic stage, the motor skill is so well-established that it can be performed automatically in a range of contexts with limited demands on attentional resources (as with the driving example above).

Shifting back into controlled mode (from automatic) can occur under a variety of circumstances (see Box 3) and with varying levels of awareness. This might be required if you were deliberately trying to reverse a habitual way of moving (for example, un-doing a bad habit you had picked up in your golf swing). Another example of shifting between these modes is when you encounter something dangerous in the environment, which requires a rapid modification of the movement, correcting the movement, the posture, and perhaps shifting back the intention. In this case, an alerting signal is provided by the system, something that may occur either without awareness or with delayed awareness. Box 4 shows how the movement program has been modified by unexpected external circumstances some time before a conscious awareness of the need to modify the movement takes place. Ullsperger et al. (2010) have speculated that we are not aware of the error-detection, but rather become aware of the subsequent arousal response in the system.

Box 4 **Automatic correction of automatic movements.**

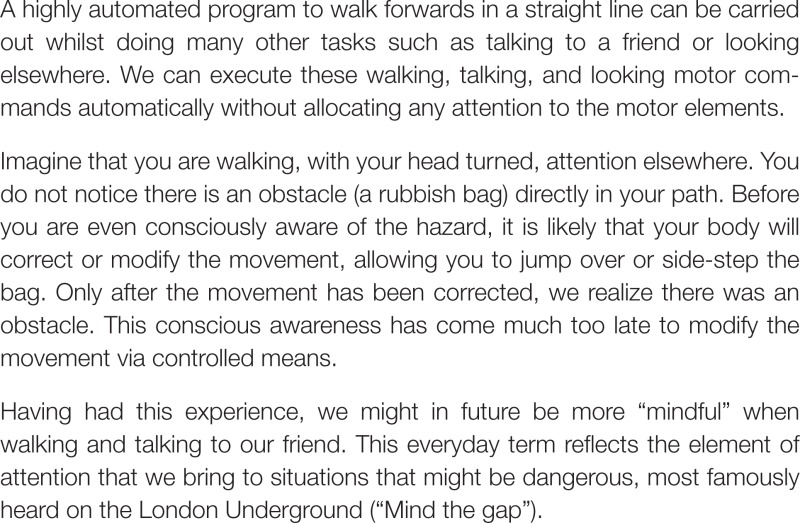

#### Prediction and Anticipation in Movement

Information processing models (combining ideas from computer sciences and observations from physiology) detail how this process of rapid correction occurs. “*It is now generally accepted that when we execute a movement we predict the sensory consequences of that movement through generative or forward models (*…*) predicted kinematics from motor commands are considered an integral part of motor execution*” (Kilner et al., 2007, p. 161). The term corollary discharge (also called “efference copy”) describes the “copy” or “template” of the predicted movement discharged into the system at the same time as the efferent motor command. This efference copy is drawn from stored memories of motor commands and their sensory consequences (Blakemore et al., 2001) and provides an internal representation of the expected sensory consequences of the movement.

The *observed* visual and proprioceptive sensations entering the system during the movement are compared against this efference copy on a moment-by-moment basis. This determines if the movement has been executed as intended (Jeannerod, 2003). If the observed sensory input (the reafferent signal) *matches* the efference copy, the movement has been conducted as planned, and there is no need to allocate attention. This process has been suggested as a way to increase the efficiency of attention and cognitive processing, by preventing the central nervous system from wasting valuable metabolic resources processing irrelevant (self-generated) sensory stimuli and maximizing the detection of the more important unanticipated or unpredicted stimuli (Pynn and DeSouza, 2013). In these latter cases (see Box 4), there is a *mismatch* between expected and observed sensory information, creating an error signal, and alerting the system to do something to modify the movement. In any movement, the mismatches are more salient. The error signal triggers a cascade of events including those that will reconfigure muscle activity, joint orientation, and velocity as well as top-down mechanisms to re-organize the movement sequence. Part of this process may involve bringing the source of the discrepancy into focus. This is an important feature of the motor system with survival value.

Environmental demands as well as changing internal goals mean we need to anticipate, predict, and process in parallel information related to our movements on a moment-by-moment basis. No matter whether the movement is well-practiced (highly automatic) or brand new, the brain creates simulations to anticipate the various stages of the movement, and the state of sensory receptors, in order to foresee possible solutions to every error, take chances, and make decisions (Berthoz, 1997).

The possibility to anticipate movements in the way described above may engage a type of simulation architecture that has been evoked in discussions of mind-wandering and the DMN. As Buckner has stated “*there may be specialized brain systems that underlie our abilities to mentally explore and anticipate future situations*” (Buckner et al., 2008, p. 31). Berthoz has also pointed to the importance of simulation in movement physiology and Jeannerod’s work proposes a simulation hypothesis of motor cognition that underpins action representation, social cognition, and language understanding (Jeannerod, 2006). Although more research is needed to clarify whether these motor and cognitive simulation systems are the same, from a philosophical point of view^6^, they appear to represent the same phenomena that “*the brain is continuously and unconsciously learning to anticipate the consequences of action or activity on itself, on the world and on other people*” (Timmermans et al., 2012, p. 1412).

In summary, motor learning models indicate how movement sequences can be highly automatic or fully conscious. Cognitive models suggest how movement commands interface with the attentional system to change our experience of an action under different conditions. Information processing models suggest how internal movement representations support flexible responding. The feedforward model is proposed to do this by predicting what we might expect to experience and then comparing this with the actual experience. In this latter system, an error-detection/alerting signal is activated when there is a mismatch that may have potential survival value. These error correction mechanisms in the movement system may rely on the same neurophysiological (LC/NA) and neuropsychological (WM) as we describe below.

### Neurophysiological aspects of movement: LC/NA

The noradrenergic system is implicated in the physiology of movements; both the autonomic aspects of movements and the more cognitive aspects.

Movement requires autonomic adjustments, such as changes in arterial blood-pressure or volume and LC–NA neuronal activity is highly sensitive to cardiovascular events (Elam et al., 1986b). Additionally, during movement, there are an increased number and variety of tactile sensations (cutaneous sensory afferents), which feed into the LC (Elam et al., 1986a,b). These afferents include those arising from the skin stretching over muscles and the sensation of the air or clothes moving across the skin. Thus, movements are likely to impact on LC–NA firing rates, via modulation of cardiovascular and sensory afferents.

Any system that monitors salience must be intimately connected to the movement system in order to ensure that the animal survives when it detects something threatening, requiring it to escape from danger (Berridge and Waterhouse, 2003). This suggestion is supported by Bortoletto’s observation (Bortoletto et al., 2011) that arousal does not directly activate structures underlying the preparation for actions, but rather influences the allocation of attentional resources to movement. As suggested in Section “Mind-Wandering and the Locus Coeruleus/Noradrenaline System,” the phasic mode of LC firing supports task-relevant information processing and selective attention. It has been suggested that the output of LC activity may “*coordinately regulate the speed and efficiency of motor responses to salient stimuli*” (Berridge and Waterhouse, 2003, p. 61). The neuroanatomical and neurophysiological properties of the LC (Berridge and Waterhouse, 2003) make it suitable for signaling the detection of unexpected state changes (Dayan and Yu, 2006) and triggering the required rapid behavioral adaptation to an environment that is constantly changing (Bouret and Sara, 2005; Dayan and Yu, 2006) (see example in Box 5). The error signal (“mismatch” in the feedforward model, described above) is a salient marker of an unexpected change in the environment, which may prompt modulation of the LC/NA system.

Box 5 **Pianist playing solo at a live classical music concert.**

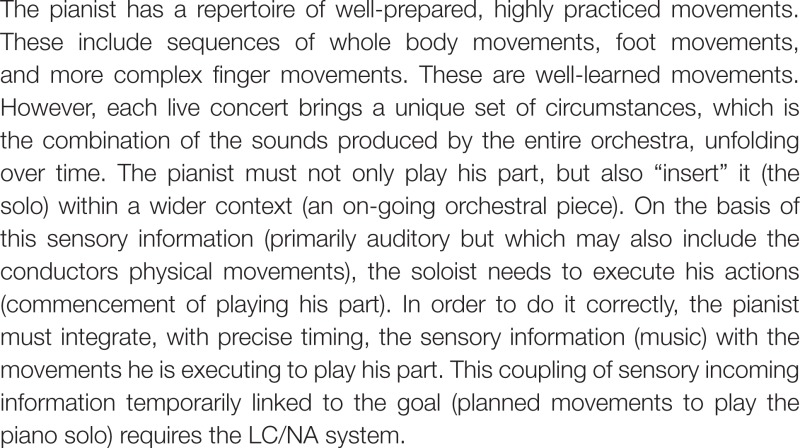

Although the exact mechanism by which the LC/NA system modulates movements has not yet been fully elucidated, it is likely an important neuromodulator, with the caveat that this system likely interacts with other neurotransmitters, such as dopamine.

### Neuropsychological aspects of movement: Working memory

The error correction process within the motor system has been suggested to imply a short-term storage of outflow information (Jeannerod, 1997), a function which could be subserved by the episodic buffer of working memory. We suggest these expected and observed sensory consequences are held, to be manipulated, within the episodic buffer system. The episodic buffer can hold multimodal information including representations drawn up from long-term memory (as would be necessary to hold all the different sorts information stored in relation to movements).

Working memory and movement programing share common processes, as indicated by studies where working memory demands have been increased, causing interference in the motor behavior. Increasing working memory load interferes with movement preparatory processes that involve cognitive control, suggesting that there is a shared resource for working memory and movement planning (Baker et al., 2011). Experiments that have explored the capacity of the VSSP under different arm movement conditions demonstrate that there is an overlap between the movement and the operation of this slave system (Quinn and Ralston, 1986). In both these studies, the effects were distinct from the effects of the experimental manipulations on attention.

Working memory is considered to play a role in the execution of motor programs that require discrete timing. Making a slow movement as we do in mindfulness requires more working memory as demonstrated by experiments showing that loading working memory interferes with slow, discrete movements more than it does with continuous, fast paced movements (Maes et al., 2015).

These findings described above would predict that movements requiring attention (engaging the WM system) will impact on mind-wandering. Teasdale et al. (1995) conducted a number of experiments to determine the relationship between working memory (specifically the CE) and a type of mind-wandering referred to as stimulus-independent thoughts (SITs). In a visuo-motor task, the production of SITs was reduced when learning the task. This effect was seen to a lesser degree once the task had been practiced. These findings are consistent with the hypothesis that the production of SITs and the control and co-ordination of movements share (and compete for) the same limit CE resources.

In terms of possible treatment implications, Teasdale et al. (1995) suggest that “*the most effective tasks to block unwanted thoughts are those that make continuous demands on the control and co-ordinating resources of the CE, but, that do not, themselves, generate SITs*” (p. 558). This suggests that new movements, requiring attention, are able to interrupt mind-wandering processes. Mind-wandering can also be reduced with mindful movements, whereby focused attention is deliberately brought to any movement, including automatic ones. This finding is congruent with reports that there is a decreased in the DMN activity (and thus a possibility to reduce mind-wandering) when working memory is increased (Koshino et al., 2014). The critical point is the engagement of attention whilst moving. This is described in more detail below.

### Mindful movements

Both movements and mindfulness engage wide-ranging brain networks that modulate arousal, activity, attention, and monitoring. Neuroimaging research suggests many overlaps between regions engaged during movement and those used for working memory and selective attention, including a fronto-parietal circuit (Harding et al., 2014) which is also activated in movement sequencing (Rushworth et al., 2001; Bengtsson et al., 2004), motor timing (Bortoletto et al., 2011), motor preparation (Collins et al., 2010), and motor learning (Jueptner et al., 1997).

Movement alone is sufficient to engage WM and the LC/NA system, due to the obvious “change” in sensory information that can be observed (e.g., posture, balance, co-ordination, speed) creating elements that can become the focus of attention. There are a number of contemplative movement practices of Eastern origin (e.g., Tai Chi, Chi Gong, Yoga) and also Western somatic education methods (e.g., Feldenkrais, Eutonia) that promote, via their somatic focus and slow speed, mindful states (a moment-by-moment felt sense of the body). These have been referred to as “movement-based contemplative practices” (Schmalzl et al., 2014). Some of these have well-recognized physical (Jahnke et al., 2010) and mental (Wang et al., 2010, 2014; Payne and Crane-Godreau, 2013) health benefits and there is a growing interest in their ability to promote mindfulness (Nedeljkovic et al., 2012).

During a slow movement, the flood of sensory information becomes apparent. It is possible to feel the air across the surface of the body through sensory receptors, the movement of the joints via proprioceptive input, and notice how autonomic responses (e.g., breathing and heart rate) are constantly being adjusted. This increased sensory information increases the perceptual load on the system (as there are more sensations to observe). The extensive literature on load theory suggests that a consequence of this is decreased distractibility (Lavie et al., 2004). The ability to process distractors is related to working memory capacity, when this is increased it enhances the ability to deal with distractors promptly. Therefore, the ability to attend, and stay attentive, may be easier during movement (see Carmody and Baer, 2008).

However, in order for a movement to be considered part of “ST-Mindfulness training,” i.e., training with the benefits of reducing stress and transforming automatic reactivity into adaptive responding, there is an additional cognitive element required. Contemplative movement practices *tend* not to deliberately engage the cognitive training elements (awareness of attention to the object or lack thereof). Specific guidance is required to bring attention to mind-wandering and the cognitive elements used to manage mind-wandering.

It is possible to do a movement and train in mindfulness simultaneously, using the moving body as the object of focused attention training. Recently mindful movement has been defined as “any movement conducted with full explicit awareness of intention, attention, and all the physical and mental sensations unfolding over time. Mindful movements are conducted with a stance of compassionate acceptance toward each and every experience including thoughts, feelings, memories, and emotions but especially bodily sensations” (Russell and Tatton-Ramos, 2014, p. 120).

When conducting a movement in mindful way, it is suggested that there may be additive effects arising from the double engagement of the systems trained in ST-Mindfulness via the movement programing and execution aspects and the focused attention/cognitive aspects, detailed in Table 2.

**Table 2:** **Distinctive guidance points between contemplative and mindful movements**.

Main mindfulness instruction	General guidance points	Contemplative movement	Mindful movement
Awareness of sensations	Bodily sensations as objects for the attention (sensations related to autonomic response, skeletal and muscular aspects, pressure, tactile and visceral, proprioception, kinematics)	√	√
Awareness of the present moment (PM)	PM attributes of body, movement sensations, and breath	Implicit	√
	PM attribute of mental experience, e.g., emphasis on staying with the present moment (not getting lost in past or future thinking)	Not usually	
Awareness of attention	Deliberate awareness of the ways in which to move, shift, narrow, and widen the focus of attention to aspects of body and movement. Commentary on the quality of the attention (vivid, dull, agitated, stable, striving)	Not usually	√
Awareness of mind-wandering	Acknowledgment and suggested management of mind-wandering	Not usually	√
Awareness of intention (on purpose)	Purposeful, deliberate engagement with the intention to move the body, pay attention, be present, mindful	Can be (e.g., Tai Chi, Feldenkrais, continuum movement)	√
Awareness of non-judgment	A deliberate attitude of acceptance and gentleness to mental and physical phenomena. For example, psychological responses to learning new movements (frustration, elation, irritation, pride)	Implicit and in relation to the physical body rather than the mind	√

A critical skill developed in mindfulness training is the ability to observe experiences (sensory information, thoughts, emotions), as they unfold. The student is asked to monitor sensations from body *and* mind, on a moment-by-moment basis. The working memory system (via CE and EB components) supports the monitoring of this constantly updated information. Similarly, the temporal ordering required to execute a movement, and process sensations coming back into the system, uses the same monitoring function and the same working memory system. Therefore, mindful movements have a dual entry point to engage the monitoring function of the working memory system (Maes et al., 2015).

Another task in mindfulness training is to hold on-line and keep track of the “intention.” The student needs to keep an active intention to pay attention, to monitor mental and physical experiences, and stay on task. For example, monitoring whether we are on task (attending to a movement) or not (thinking about dinner). There is a parallel process in the movement system. Jeannerod (2003) proposed that movement “*requires not only the simulation of the whole action and its consequence in the external world, but also the monitoring of the intention-related signals*” (p. 162). Movements require the on-going monitoring of the efferent motor commands and afferent sensory signals to ensure the movement is going as intended (Blakemore and Decety, 2001). Therefore, intentionality is embedded within any volitional movement (Jeannerod, 2003). Here, we again see that mindful movements may have a dual entry point into the working memory system via the monitoring of the intention.

## Clinical Populations

In the state of mind-wandering, Berthoz and Petit have suggested that *“rather than fixing its gaze in the facts of the world, the subject is focused on mere representations. All mental states are already there, peacefully juxtaposed along the one and only homogenous plane of ‘Mind’ ”* (Berthoz, 1997, p. 14).

Although it has been suggested that mind-wandering may have an adaptive function in the pursuit of long-term goals (Smallwood and Andrews-Hanna, 2013), this system can be engaged in maladaptive/pathological ways. In the clinical setting, extreme mind-wandering in the form of ruminations and recriminations is not peaceful, and can be experienced as distressing. We have discussed how modulation of LC firing is implicated in mind-wandering. The involvement of this system in psychiatric conditions is suggested by the clinical picture of many disorders (Yamamoto and Hornykiewicz, 2004; Yamamoto et al., 2014). For example, in individuals with bipolar, there are high levels of arousal and anxiety and correspondingly high distractibility and poor attention (Thompson et al., 2005; Kung et al., 2010). In generalized anxiety states, the ability to imagine and rehearse future scenarios is over-used and sometimes overwhelming (anticipatory anxiety). In depression, firing rates are low, leading to the clinical signs of reduced physical activity. Most psychiatric conditions have some pattern of disrupted sleep supporting the notion of LC abnormalities. In Table 1, a summary of the different modes of LC firing is shown with reference to what occurs in patient populations.

For a clinician interacting with psychotic patients, it is often difficult to define whether their experience is, at that moment, a hallucination (aberrant sensory processing), a delusion (aberrant thought processing), or both. At the physiological level, the boundaries between perception and belief are considered to be less distinct, with both being dependent on prediction. Confusion can arise when there is a failure to update inferences and beliefs about the world, arising from a discrepancy between predicted and observed sensations. This has been offered as an account of hallucinations and delusions (Fletcher and Frith, 2009). In the motor domain, this manifests as passivity phenomena (where self-generated actions are attributed to others). Reinforcing the importance of the brain’s capacity to predict and compare constantly updated information, Berthoz, who has worked primarily in the field of locomotion physiology, suggests hallucinations may be a type of “waking dream,” in which internal circuits that are used to simulate the consequences of action are functioning autonomously (Berthoz, 1997). Clinically, this corresponds to the observation of patients in psychotic states who appear to be living as if in an “internal scene.”

This reality distortion (Liddle, 1987) may be due to a disturbance in the capacity of the brain to simulate and hold on-line a version of reality, with a failure to update information at a suitable pace and with a specific temporal order. The confusion between perceptions resultant from imagined scenarios and those from the external world may arise from over-activity in DMN (Buckner et al., 2008). Abnormal functional and structural connectivity between brain regions are considered a core feature of schizophrenia, with hyper-connectivity in the DMN seen in these patients (Whitfield-Gabrieli and Ford, 2012). This hyper-connectivity has been proposed to result in a propensity to be overly self-referential, poor cognition (working memory, executive functions), and poor social cognitive performance (Buckner et al., 2008; Spreng et al., 2009). The latter includes the ability to simulate or hold “in mind” the mental states of others, a critical skill for social interactions, known as theory of mind.

Below, two key neurophysiological and neuropsychological deficits seen in psychotic patients are further explored to provide an understanding of why mindful movements are helpful when working with this population and why adaptations are needed (the latter detailed in Section “Clinical Application of Mindful Movement: A Description of a Practical Experience with Psychotic Patients”).

### Impaired LC/NA and WM functioning in patients

Patients with schizophrenia are known to be highly distracted (Chapman, 1956; Hemsley, 1976) and with defective filtering mechanisms (Saccuzzo and Braff, 1986). These impairments in information processing may reflect disturbances in the noradrenergic system. Although a number of neurotransmitters are implicated in the pathophysiology of schizophrenia, there is growing evidence that disruption in the noradrenergic system is a key contributor (Lieberman and Koreen, 1993; Yamamoto and Hornykiewicz, 2004; Craven et al., 2005; Lechin and van der Dijs, 2005). There are correlations between increased levels of NA and relapse in these patients (Van Kammen, 1991), which appear to be independent of medication effects (Van Kammen et al., 2014). Also, evidence has been found for increased central NA output (Friedman et al., 1999), elevated cerebral spinal fluid-NA associated with states of over-arousal (Kemali et al., 1990), and dysfunction of NA receptors in pre-frontal cortex associated with cognitive impairments in these patients (Friedman et al., 1999).

As discussed previously, modulation of the LC/NA system is associated with different ways to process incoming sensory information. Thus, high tonic levels of LC firing are associated with decoupling of the NA bursts from salient stimuli, resulting in poor discriminability and increased distractibility (Smallwood et al., 2012). It has been suggested that boosting of task-irrelevant signals (high tonic mode) increases “the conscious expression of intentional thoughts that are not directly related to the current task” (Smallwood et al., 2012, p. 2). With this firing pattern, there is a reduction in perceptual input from external sources, and the individual may be absorbed in their own internal experience. This description corresponds to clinical observations of psychotic patients, lost in their own mental experience, who are also sometimes incapable of determining whether information is arising from an internal or external source (as is the case with hallucinations). Thus, one interpretation is that the natural tendency to mind-wander is amplified in these patients due to the high tonic firing rate of the LC. Furthermore, abnormal P300 (also called P3) has been described as one of the most robust markers of schizophrenia (McCarley et al., 1993). P300 is an evoked potential signal that occurs after novel and task-relevant stimuli have been processed. It is considered an electrophysiological correlate of the LC phasic response mode (Nieuwenhuis et al., 2005). These findings point to defective phasic modulation of NA in the LC in schizophrenia, in addition to the tonic abnormalities.

From a neuropsychological perspective, working memory deficits are a key feature of schizophrenia (Goldman-Rakic, 1994; Forbes et al., 2009) and a target for treatment (Lett et al., 2015). Working memory difficulties are even more pronounced in those with formal thought disorder, who show particularly striking decrements on tasks assessing both VSSP and verbal components of the working memory system (Arcuri, 2003; Arcuri et al., 2010). Clinicians struggle to support these patients adequately, as psychological treatments often require the very cognitive abilities that are impaired. This may also preclude ST-Mindfulness, as these practices require working memory.

### Making the case for training mindfulness with patients using mindful movement

In the previous sections, we have described two mechanisms, one neurophysiological (LC/NA system) and one neuropsychological (WM) implicated in the ST-Mindfulness training (and focused attention training). We have also described the involvement of these two mechanisms in movements, and particularly in mindful movements. We further pointed to evidence of impairment of these same mechanisms in psychotic patients, and the resultant implications for using ST-Mindfulness static practices with these groups. On this basis, it is clear why ST-Mindfulness may be difficult and/or unsuitable for patients with psychosis. These observations can help inform theory-driven adaptations for the delivery of mindfulness training, allowing these individuals to benefit from mindfulness, delivered via another route: mindful movement. Before we describe our experience of doing this work, we summarize the rationale for why we believe this method works.

In psychosis, there is an extreme level of mind-wandering (patients are highly distracted). When using movements as the object of attentional training for mindfulness, you are engaging the same brain architecture (anticipation/simulation) as is engaged in mind-wandering states. This, we suggest, leaves less room for positive symptoms as the mind-wandering “machinery” is occupied with movement preparation and execution. At the neural level, mindful movements in those with high distractibility possibly facilitates the switching away from the DMN and toward attentional/executive and SNs.

Awareness generally is problematic for these patients, most likely due to impairments in the CE/EB system. The concrete, physical sense of movements and their timing properties (a beginning, middle, and end of a movement) provide strong anchors against which to detect mind-wandering. It is easier to detect the difference between a concrete and an abstract sensations (movement sensation versus an inner voice) as compared to two abstract sensations (a thought and an inner voice). With respect to the timing aspect, it is easy to become aware that your mind has wandered off during a movement because a very obvious portion of a defined movement sequence would have been completely missed (escaped your attention) while you were “gone.” With supportive guidance from the facilitator (shown in Table 3), awareness of general mind-wandering (e.g., thoughts about dinner) and symptom-specific mind-wandering (e.g., auditory hallucinations) can be trained. The differentiation between the target of attention (movement) and the distractors (“normal” mind-wandering and/or the more extreme symptom-specific mind-wandering) is made easier as it does not rely solely on engagement and monitoring of the internal world.

**Table 3:** **Guidance for delivery of mindful movement for psychotic patients**.

Main mindfulness instruction	Specific guidance modifications with patients
Awareness of sensations	Increased prompts due to poor attention and high distractibility
	Increased support for voluntary attentional shifts (‘just keep bringing attention back to the movement”)
	More specific guidance about the types of sensations that may be attended to
	Encouragement of self-generated alterations to movements and their sensory consequences
Awareness of the present moment (pm)	Ask to keep checking if the sensation they feel is the same moment-by-moment
	Exploring the suggestion that no two movements are the same
	Reminders that each movement is a brand new “present moment”
	Exploring the temporal qualities (beginning, middle, and end of movements) and pacing of the movement sequences
Awareness of attention	Acknowledgment of increased mind-wandering
	Acknowledgment of the effort required, and that this will improve with practice (using the gym/muscle training analogy)
	Repeated reminders to monitor where the attention is at any given moment
Awareness of mind-wandering	Indicate that mind-wandering is normal
	Treat all mental experiences as equivalent to physical sensations (including “abnormal” or distressing mind-wandering such as voices, imagery etc.)
	Point to categories and types of mind-wandering
Awareness of intention (on purpose)	Repeated reminders about the intention to attend
	Reminders about why this practice is helpful
	Prompts to attend to the intention to move
Awareness of non-judgment	Repeated reminders about the intention to be gentle with physical and mental experiences
	Reminders to be gentle in response to distressing symptoms
	Supporting and encouraging any attempt at a movement (no right or wrong way to move)
	Reminders to be gentle with self and others

In psychotic patients, WM and LC abnormalities may present as a difficulty in holding on-line the intention to pay attention, holding on-line the intention to monitor, shift, and direct attention, and problems with goal-directed movements. When completing slow mindful movements, it is easier to shift attention back to the movement because it is being conducted in a controlled way (which requires attention) rather than automatically. In terms of the intentional shift back to the object, in mindfulness training, this is usually held “in mind” by the individual, but in psychotic patients this needs to be done via guidance. The guidance of the facilitator supports the working memory deficits (perhaps functioning as an external, auxiliary, working memory system).

Psychotic patients have high distractibility and problems in maintaining a sustained, focused attention on any object. It has been suggested above that this may be related to the reported abnormalities in the LC/NA system. Specifically, we suggested that high tonic LC firing rates are linked to high levels of arousal (which makes paying attention difficult) as well as phasic firing mode disruption (making selective focused attention problematic). These difficulties can be mitigated with the correct preparation of the environment and individual prior to practice and by specific guidance to support attention from the facilitator. The moving body provides rich (and perhaps novel) sensations to support focused attention and reduce distractibility (as proposed by load theory).

### Clinical application of mindful movement: A description of a practical experience with psychotic patients

In the following sections, we share our experience of working with adults with severe and enduring mental health conditions, offering mindful movement classes to inpatients in various settings. These observations arise from work on the development of a mindful movement protocol called Body in Mind Training (BMT; Russell, 2011; Russell and Tatton-Ramos, 2014) designed by one of the authors (TR, see Box 6). BMT uses a series of movements, many of which are derived from tai chi, performed with directed attention. This training has been developed primarily in the mental health setting, both with acutely ill and more stabilized community patients.

Box 6 **BMT class structure.**

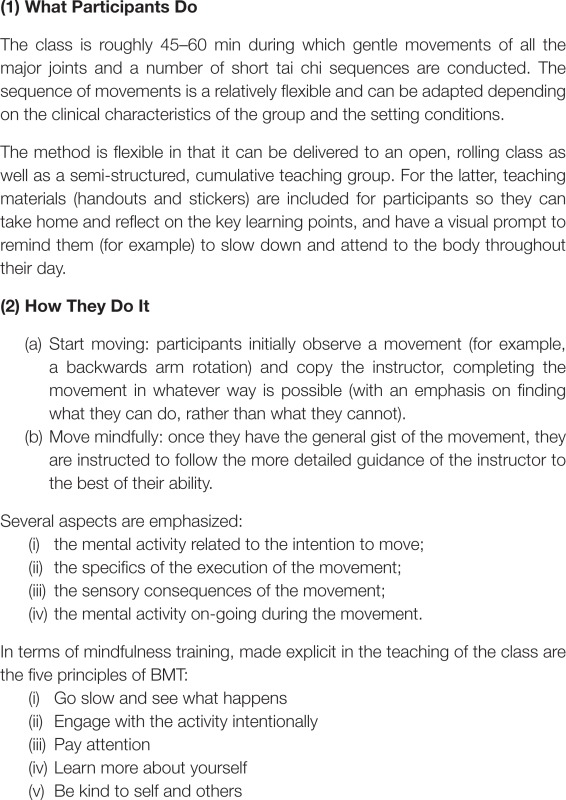

There are three aspects of what happens in this class, which may explain the beneficial effects observed. One is the care taken to support a down-ward shift in the tonic levels of arousal (from high to low). The second is the use of slow body movements, engaging (and competing with) the same simulating system as is engaged in mind-wandering. The third is the guidance of the facilitator, providing an auxiliary working memory system (holding intention, altering the mind to mind-wandering, guiding them back “on task”).

#### Creating Environmental and Psychological Conditions to Reduce Arousal

Individuals in psychotic states are suspicious, frightened, feel persecuted, and their sensory perception is distorted (e.g., hallucinations). Table 1 shows the underlying neurophysiology of these high levels of arousal. Extra care is therefore needed to create a suitable physical and psychological space for this work. In terms of environment, these groups are best delivered in a relatively quiet and well-ventilated area, ideally off the ward. A gym environment is ideal as it helps to frame this training as something one might do in everyday life (rather than being seen as a “treatment”), it encourages those who might be reluctant to attend a “mindfulness” or “meditation” group, and may increase the chance that these practices are continued following discharge from hospital.

Participants have commented that music helps them to settle when coming from busy ward/urban environments, and additionally that it helps to mask some of the distracting environmental sounds. Although typically music is not used in mindfulness classes, in this setting, it can help reduce arousal levels by serving as a noise filter. Music might be turned off halfway through the class in order to deliberately explore what happens in the mind (to attention and mind-wandering) when there are more distractions.

From a psychological point of view, the instruction in ST-Mindfulness to sit still, in silence and with eyes closed, is not ideal for psychotic patients and may even increase distress. The requirement to copy the facilitator’s movements requires visual attention, so eyes must (initially at least) be open. Encouraging the spirit of curiosity and experimentation, participants can be invited to experiment with eyes open or closed, if they feel comfortable to do so.

In mindfulness training, a critical step is the personal decision to choose to engage with mental and physical experiences in a different way. In these classes therefore, there is a chance to explore personal responsibility and engagement with self and one’s actions. Participation is explicitly voluntary, with each participant responsible for their own level of engagement. This may provide a rare therapeutic opportunity for autonomy. Participants can lie down, sit down, take a break, and regulate their involvement; the only rule is to be considerate of others in the class. There is thus a high degree of flexibility in delivery, which allows participants to find their own way to start moving mindfully. Modeling gentleness in every instruction and response is extremely important to counteract the aggression these individuals are experiencing internally (e.g., the negative and derogatory comments in auditory hallucinations).

#### Learning and Performing Movement Sequences

In BMT, some of the movements may be familiar (for example, a “swimming backwards” arm movement) while others might be new sequences of movements (moves adapted from Tai Chi such as “wave hands like clouds”). New movements require engagement of working memory and attentional focus (as described in Section “Movements”). When introducing new movements, there needs to be enough challenge without being overwhelming. Scaffolded learning methods (Young et al., 2002) can be used, with the facilitator providing “step down” options if the full movement sequence is not possible for either cognitive or physical reasons. Participants are encouraged to modify, adapt, slow down, or do whatever is necessary to get into the sequence, but to keep on trying, with each movement a new opportunity to start again. An unexpected observation in these classes was the way self-efficacy was developed when participants supported each other to learn new sequences. During the learning phase, the non-judging component of mindfulness needs to be strongly emphasized.

The transition between movement sequences tends to be much quicker, in order to combat the increased potential for mind-wandering and hold the attentional focus. Table 3 shows some specific guidance points related to holding attention on sensations and working with mind-wandering. New and familiar movements may be interwoven throughout the class and new movements revisited to provide experiences of different levels of mental effort (and a chance to notice different types of mental reactivity).

Some participants may not be able to do the movements slowly, so they are invited to play with the pacing of the movements. Changing the speed brings automatic movements into the controlled mode and results in different kinematics and sensory experiences. Optimally, movements are conducted slowly and gracefully, however the main invitation is to notice how sensations can be different at different speeds.

#### Specific Guidance

Participants are first invited to copy the movement of the instructor in order to get the general gist of the movement. Copying movements may reduce the cognitive demands engaged in planning and executing movement via automatic engagement of the mirror neuron system, known to be activated when we observe others moving (Rizzolatti et al., 2009).

Participants are additionally given specific, frequent verbal guidance. This guidance supports the dysfunctional working memory by alleviating the requirement for the patient to hold their intention (to move, to attend, to shift, and re-orient attention to the body) in their working memory system. The use of frequent verbal prompts helps to orient attention and the moving body is the object for the attention. In this way, “top-down” cognitive support is provided (see also Chadwick, 2014).

Suggested guidance modifications are shown in Table 3 and are framed around Kabat-Zinn’s definition of mindfulness. The guidance points to what you might attend to in the movement [including sensations from the body, movement features as well as the things that might capture attention (internal/external distractions)]. This allows a detailed description of bodily sensations and movement processes alongside guidance that helps participants to see it is possible to hear a voice or experience an image, and without denying or fighting it, gently moving their attention to the body and returning to the present moment. Prompting an adjustment of posture or suggesting a modification to the movement helps to re-orient the individuals” attention and de-couple from the mind-wandering.

A specific difference between this training for these patients in a mental health setting and what might occur in a “community” contemplative movement class, is the acknowledgment within the guidance of the mental experience of these participants (rapid thoughts, high arousal/anxiety, disorganized thinking, and distressing imagery or voices). Developing this type of strategy to work with positive symptoms like auditory hallucinations has been suggested as a key intervention (Shergill et al., 1998). For example, saying “noticing if the mind is distracted by internal dialog, voices, or imagery and without judging that experience, trying your best to come back to the sensation of the movement of the shoulder blade, noticing the speed, the effort, any places of tightness or ease.” This instruction is much more detailed in comparison to a more traditional guidance, which might say invite participants to “just notice the mind-wandering and bring it back to the body.”

In summary, these clinical observations of the delivery of mindful movement to psychotic patients speak to the face validity of the theoretical ideas. The *“how”* of these mindful movement sessions is different from a typical contemplative movement class; the delivery is adapted, the movements are adapted, and the modified guidance is critical. It has been suggested that delivery of mindfulness in any format to individuals with severe and enduring mental health conditions requires a facilitator who is experienced not only with mindfulness, but additionally with psychosis and psychological therapy (Chadwick, 2014). In BMT, movement experience is also required.

## Discussion

### Summary

Based on the literature, this article has highlighted some candidate processes (neurophysiological and neuropsychological) that are likely implicated in mindfulness training using movements. A case for an essential role of the LC/NA system and working memory (particularly the CE and the episodic buffer components) has been made. We hypothesized that these components may be directly involved in maintaining the focus of attention within intended goals and staying within the experience, as it unfolds moment-by-moment. We described evidence from the literature that the moving body also engages the same neurophysiological and neuropsychological systems. A distinction was made between contemplative movements and mindfulness movements on the basis of the explicit training of attention and insight into mental experiences (e.g., mind-wandering) that occurs in the latter. Below, we draw together evidence from the neuroimaging literature to support the suggestion that the distinct phenomena of “meditating,” “moving,” and “experiencing psychotic symptoms” are associated with activity in certain (similar) brain networks (including the DMN, the SN, and an attentional/executive network). We propose that training with mindful movements engages these networks via a different route, one that is possible for those with schizophrenia, as detailed in our clinical observations (Section “Clinical Application of Mindful Movement: A Description of a Practical Experience with Psychotic Patients”).

We have described the role of the DMN in mind-wandering and how activity in the DMN activity can be diminished via meditation. DMN activity may also be reduced when working memory load is increased (Koshino et al., 2014). Working memory load is increased when planning and executing movements. Therefore, mindful movement is likely to be associated with reduced activity in the DMN. Engaging working memory via mindful movement also places demands on the attentional/executive and SNs. Shifting between these networks (states of mind-wandering versus attending to movement) is thus required when moving in a mindful way. For this reason, the combination of movements with mindful attention meditation training may be particularly effective to reduce mind-wandering, whilst training the focus of attention.

The ability to engage and disengage DMN, SN, and the attentional/executive networks may involve phasic activation of the LC/NA system, to facilitate flexible responding in the face of ever-changing environmental conditions (Bouret and Sara, 2005). In schizophrenia, the LC/NA system is dysfunctional, and there is evidence of impairment in these three networks (Palaniyappan and Liddle, 2012; Whitfield-Gabrieli and Ford, 2012; Orellana and Slachevsky, 2013). Poor switching between internal and external foci of attention may also contribute to the observed cognitive impairments (Whitfield-Gabrieli and Ford, 2012). We thus suggest that impairments in flexible and adaptive responding, requiring the integration of stimuli from internal and external sources, via modulation of these three networks (DMN, attentional/executive networks, and SN) is impaired in psychotic patients and may arise from LC/NA system deficits. Palaniyappan and Liddle (2012) have suggested that the SN has a role pivotal to shifting between these networks. However, our clinical experience shows that mindful movement may, with modifications, be able to mitigate the impact of these impairments and provide a route to mindfulness training.

Hyper-connectivity in the DMN (described in Section “Clinical Populations”) has also been associated with the cognitive abnormalities and positive symptoms seen in schizophrenia. These patients are highly distractible and lost in their inner world, paying little attention to external sensory information. They also have impaired working memory. For these reasons, mindfulness training needs to be adapted, in a way that takes into account the underlying disrupted mechanisms (and consequent impaired performance). We have spoken about the extra guidance requirements to support this training (Table 3 and Section “Specific Guidance”). The additional guidance operates as a top-down support for movement planning and execution. This may partially compensate for executive functioning deficits in these patients (operating as an auxiliary working memory system) and modulate the attentional/executive network. Similar methods (directing attention to the relevant features to be observed) have been used to improve performance on a facial emotion recognition task in schizophrenia (Russell et al., 2008; Marsh et al., 2010).

The hyper-connectivity in DMN is also associated with prob­lems inferring the mental states of others (theory of mind). Theory of mind develops from the natural capacity of the brain to simulate the intentions of others on the basis of observed movement (Blakemore and Decety, 2001), and is impaired in schizophrenia (Russell et al., 2006). This simulation system, associated with the DMN (Buckner et al., 2008; Spreng et al., 2009) may thus be engaged during mindful movement as participants observe and copy intentional movements. So, in addition to the top-down support provided, the requirement to observe and copy movements may offer a different (bottom-up) entry point into these same systems.

### Possible research

There remain however, a number of outstanding questions, which may provide avenues for future research. Investigations may be conducted at a variety of levels of explanation (neurophysiological, neuropsychological, clinical).

At the level of neurophysiology, one research avenue would be to test more formally whether mindfulness training will change the tonic and/or phasic firing rates of the LC/NA system. A second avenue might be to explore whether mindful movement training will impact on the DMN and/or other network activity in a way that is different from ST-Mindfulness. Static and movement-based interventions matched for every aspect by except movement might be compared in healthy participants. Distinctions were also made between contemplative and mindful movement trainings and these could also be compared, both at the neurophysiological and psychological levels.

From a psychological point of view, we have touched on a number of theories that may explain why movements provide a potent attentional focus. Incoming sensory information from the body during movement is different from that experienced during static practices. Using load theory as a framework (in both healthy and psychiatric patients), the differential effect on distractibility could be tested.

Baddeley’s model could provide a framework to explore the benefits of mindfulness training on working memory. Tests of working memory (and its different sub-components) could be administered to meditators of different levels of experience (and including those who may come from contemplative movement traditions, or who have been trained in mindful movement). Tests of working memory could also be administered to patient groups who are undergoing different types of mindfulness training (movement, non-movement) to determine the malleability of this cognitive impairment and determine if there is any enduring effect of the training. Baddeley’s model, although suggested to process multimodal representations, does not explicitly refer to multisensory information coming from internal sources (e.g., the body); it is not clear how sensory information from the body gets into the working memory system and/or is transformed into a higher order representations (Quak et al., 2015).

From a clinical point of view, given the link between NA, stress, and relapse, another avenue might be to determine whether a mindful movement program can help to prevent relapse (monitoring admissions or clinic visits for example) or increase compliance in other therapies (perhaps via an adjunctive effect). Furthermore, given that the illness of schizophrenia includes motor abnormalities, a further line of research might look at changes in measures of gait and movement kinetics following training in the deliberate, mindful engagement with the movement process.

Finally, as with ST-Mindfulness training, the effects of being in a group, sharing experiences, the experience of the facilitator and the non-judgmental space in which to explore the self are all likely contributors to the experience. In order to determine which of these factors are essential to any observed benefits, and delineate the relative contribution of content and process, dismantling studies would need to be conducted (Williams et al., 2014). These types of studies compare tightly controlled conditions to tease out the relative contribution of each of the components (for example, offering movements alone without sharing of experience, varying the experience level of the facilitator, or offering the training in a group versus individual format). In order to measure the efficacy of any mindful movement program, it will be necessary to tease apart the effect of movement, mindfulness, and then the combined effect of moving mindfully.

## Limitations

One limitation in this work is the ability to measure accurately changes in the felt sense of the body (Mehling et al., 2009). Although mindfulness training increases the window of consciousness with respect to movement, there are still core impenetrable elements of movement that lie well out of the reach of awareness (as described in Box 4). The measurement of body awareness is highly problematic in healthy participants, and perhaps even more so in psychiatric populations. However, this does not preclude measurement of clinical and/or cognitive outcomes following mindfulness training.

Another important issue is how best to address the flexibility inherent in the delivery, and whilst still offering an effective and replicable outcome. Measures of both physical and cognitive change may be useful in this respect and help to disentangle aspects that are core to the efficacy from those that might be interchangeable.

## Conclusion

Ultimately mindfulness training is a way to help manage more skillfully no matter what the experience. Therefore, as is the case in the wider mindfulness research field, an alternative strategy is to find out what is helpful about these interventions from participants directly, gathering qualitative information to understand what is really making the difference in their lives as a result of these practices. We end with an anecdote from a participant who suffers from schizophrenia who had been attending a drop-in mindfulness movement class. After practicing mindful walking, he was able, even when experiencing paranoid thoughts, to leave his home and go to the shop to buy milk to make a cup of tea. On that short journey, he maintained his attention on his feet, and his body as he moved, step by step, and managed to do something that previously would have been impossible. Even such a small thing that most of us would take for granted, can make a huge difference for these clients. Our intention with this article is to stimulate ideas and research that can continue this work.

## Conflict of Interest Statement

Tamara Anne Russell is the creator of the Body in Mind Training Program, which is a group mindfulness training program offered in the private sector. Silvia Maria Arcuri declares that the research was conducted in the absence of any commercial or financial relationships that could be construed as a potential conflict of interest.

## Footnotes

^1^Including mindfulness-based stress reduction (MBSR) and mindfulness-based cognitive therapy (MBCT).

^2^The default mode network brain regions include four main areas: medial pre-frontal cortex, posterior cingulate and retrosplenial cortex, left and right inferior parietal lobule, and an additional region, the medial temporal lobe.

^3^The locus coeruleus, a nucleus in the pons (part of the brainstem) is the principal site for brain synthesis of noradrenaline.

^4^Also referred to as norepinephrine.

^5^The VSSP integrates visuospatial information from multiple sources including visual, tactile, and kinesthetic, and from both episodic and semantic long-term memory.

^6^This is a large philosophical area of debate encompassing enacted cognition and embodiment that is outside the scope of this paper.
